# Fertility and digital technology: narratives of using smartphone app ‘Natural Cycles’ while trying to conceive

**DOI:** 10.1111/1467-9566.13199

**Published:** 2020-11-04

**Authors:** Pippa Grenfell, Nerissa Tilouche, Jill Shawe, Rebecca S. French

**Affiliations:** ^1^ Department of Public Health, Environments & Society London School of Hygiene and Tropical Medicine London UK; ^2^ School of Nursing and Midwifery University of Plymouth Plymouth UK

**Keywords:** Fertility awareness, conception, digital technology, science and technology studies, qualitative research, UK

## Abstract

Fertility awareness apps, which help to identify the ‘fertile window’ when conception is most likely, have been hailed as ‘*revolutionising*’ women’s reproductive health. Despite rapidly growing popularity, little research has explored how people use these apps when trying to conceive and what these apps mean to them. We draw on in‐depth, qualitative interviews, adopting a critical digital health studies lens (a sub‐field of science and technology studies), to explore the experiences of cisgender women and partners with one such app, *Natural Cycles*, in the context of their daily lives. We found that many women valued the technology as a ‘*natural*’, inobtrusive alternative to biomedical intervention, and a means of controlling and knowing their bodies, amid a dearth of fertility‐related education and care. Yet this technology also intervened materially and *affectively* into the spaces of their lives and relationships and privileged disembodied metrics (temperature) over embodied knowledge. Meanwhile, app language, advertising and cost have contributed to characterising ‘*typical*’ users as white, heterosexual, affluent, cisgender women without disabilities. In the context of neoliberal shifts towards bodily self‐tracking, technologies appealing as novel, liberating and ‘*natural*’ to individuals who can access them may nevertheless reproduce highly gendered reproductive responsibilities, anxieties and broader health and social inequalities.

## 1 Introduction

Within a fast‐growing ‘Fem Tech’ industry, fertility awareness smartphone apps are among the most popular health‐tracking technologies in high‐income countries and are gaining popularity around the world (Haile *et al*., 2018, Lupton, 2015b, Moglia *et al*., 2016). Such apps help identify the ‘fertile window’ within the menstrual cycle when conception is most likely, based on a wide range of personal information, such as users’ mood, sexual and physical activity, bodily signs, symptoms and dimensions. Two indicators frequently used are daily basal body temperature (BBT) (reached after at least three hours of rest), which retrospectively indicates when ovulation occurred, and cervical secretions, which become wet, clear and stretchy at ovulation (Freis *et al*., 2018). In the UK, over a third of cisgender women using fertility awareness apps do so to support conception (Gambier‐Ross *et al*., 2018).

In public health and popular discourse, it is often argued that such technologies afford greater bodily autonomy (Moglia *et al*., 2016) and indeed ‘*revolutionise birth control*’ (Savage, 2017). Yet social scientists argue that they also place responsibility on women to be good, ‘*digitised reproductive citizens*’ who ‘*master*’ their fertility and maximise their (and their prospective child’s) health through self‐tracking (Lupton, 2015a). They have raised concerns over data privacy and commodification (Lupton, 2017), and, in effect, the exclusion of economically and socially marginalised groups (Lupton and Maslen, 2019), casting doubt over the emancipatory potential of technologies that are the product of existing social, political and economic systems (Lupton, 2016). Sexual and reproductive health‐related apps often reproduce normative gendered assumptions, with apps monitoring sexual pleasure and performance aimed at men, and those tracking fertility, pregnancy and parenting aimed at women (Lupton, 2015b), with little consideration of sexual and gender diversity. Although apps designed for prospective fathers offer valuable information and encourage them to support partners, they tend to ‘*condescend…and trivialize their role’* (Thomas *et al*., 2018: 759). Furthermore, evaluations have critiqued the limited evidence‐base and accuracy of such apps (Freis *et al*., 2018).

Studies in Sweden (Chen, 2017), the UK (Gambier‐Ross *et al*., 2018) and Australia (Lupton, 2017) indicate that cisgender women value fertility awareness and pregnancy apps and related online resources, as sources of knowledge, control, reassurance and peer support. In the UK, women appreciated constant access via their smartphones, ease of entering data, apps’ medical grounding and accuracy (Gambier‐Ross *et al*., 2018). They were generally unaware or unconcerned around use of their data to commercial ends, in common with women in Australia using pregnancy and parenting‐related apps (Gambier‐Ross *et al*., 2018, Lupton, 2017). Yet just two participants in the UK study were using apps to aid conception, among a mostly middle‐class, white, heterosexual sample (Gambier‐Ross *et al*., 2018). We know of no qualitative studies focused solely on this topic, nor any that include partners’ perspectives, reflective of tendencies in broader reproductive health research (Thomas *et al*., 2018).

The fertility awareness app *Natural Cycles*, which reports over a million users, has received considerable media attention. Originally developed in Sweden by two physicists who are married to each other, the app has since been rolled out commercially in a number of countries, including the UK. It uses an undisclosed algorithm to calculate the fertile window, allowing for uncertainties in pre‐ and post‐ovulation temperatures and ovulation day predictions. Users pay an annual subscription (£40 at the time of research), receive a BBT thermometer, and can select from three modes, to ‘prevent’, ‘plan’ or ‘follow’ a pregnancy. They enter daily BBT readings and details of their menstrual cycle and are encouraged to purchase luteinising hormone tests to predict ovulation (‘ovulation strips’). Uncertainty is reduced, and fewer ‘fertile days’ indicated, as they enter data on more cycles. In ‘plan’ mode, a green icon denotes ‘infertile’ days and a red icon darkens to indicate increasing fertility; temperature data and ovulation predictions can also be viewed graphically. *Natural Cycles* is thus far the only app to receive CE certification and FDA approval as a contraceptive, but the company received criticism for using perfect rather than typical use contraceptive effectiveness rates in its initial advertising (Hough and Bryce, 2019). To date, media reports (e.g. Sudjic, 2018), the company’s advertising and research (Berglund Scherwitzl *et al*., 2016, Chen, 2017) have centred on contraception, largely in Sweden, with little focus on how people use the app when trying to conceive.

In this paper, we draw on critical digital health studies, a sub‐field of science and technology studies first described by Lupton (2014) as work that researches and theorises *‘the social, cultural, political and ethical dimensions of the digital health phenomenon*’. Critical digital health studies help us to understand technologies, widely adopted in medical and public health domains, ‘*not as neutral, value‐free devices and software, but as sociocultural artefacts…invested with tacit assumptions and cultural meanings*’ (Lupton, 2016: 4). As well as recognising the power of industry, the medicalisation of health and digital inequalities, it involves exploring ‘*the dynamic nature of people’s interactions with technologies’* – with attention to subjectivity, embodiment and affect – ‘*in a world in which the digital is increasingly part of everyday lives, social relationships*’ (Lupton, 2016: 4). The field draws on concepts of *biopower* and *governmentality*, which respectively describe increasing systems of surveillance over (women’s) bodies (Foucault, 1978) and concurrent expectations that individuals educate and protect themselves with personal responsibility and self‐care as state provision is rolled back (Foucault, 1991). We explore socially and materially embedded *health practices* to consider interactions between individuals, objects, bodies, social actors and systems, and linked power relations (Cohn, 2014). We examine how participants *navigate* fertility (van der Sijpt, 2014), ‘choice’ and ‘control’, paying attention to bodily and social processes, norms, ambiguities and contradictions (Earle, 2004).

In this research (The Freyja Study), our aim was to explore the role of *Natural Cycles* in relation to users’ and partners’ (pre‐)conception practices and experiences. We explored how people used this and other methods when trying to conceive, how they felt about them, and how they fitted in with their everyday life and plans.

## 2 Methods

Between January and April 2019, we carried out individual in‐depth interviews with 30 people who had ever used *Natural Cycles* in ‘plan’ mode (n = 24), or whose partner had done so (n = 6). Eligible‘users’ were aged 18–44, had experience using ‘plan’ (currently or previously), lived in the UK, had not been advised to avoid pregnancy for health reasons and spoke sufficient English to participate in an interview. Partners of users who met these criteria, aged 18 or above, were eligible. Partners interviewed were not necessarily partners of users interviewed.

We recruited participants via an in‐app message posted to potentially eligible users by *Natural Cycles* (n = 23), through study social media accounts (Twitter, Facebook, and by contacting the *Natural Cycles* Facebook user group) and by asking some participants to invite their partner to participate (‘snowball’ sampling) (n = 7). A website (https://www.lshtm.ac.uk/research/centres‐projects‐groups/freyja‐study) hosted an introductory video, study information and a short series of questions (*Online Surveys* software) for potential participants to complete, with consent, to determine eligibility and aid purposive sampling (see below). Those eligible were asked to provide their name and contact details and informed that a researcher may contact them. We received responses from 344 users and 13 partners.

We purposively selected users to reflect maximum diversity in age, ethnicity, partner gender, relationship status, employment status, income‐coping, duration using *Natural Cycles* in ‘plan’ and prior pregnancy. We aimed to do the same for partners, in relation to age, gender, employment status and income‐coping. We were not able to sample for gender diversity among users because, although we purposefully used non‐gendered language, our sampling survey did not ask users about their own gender identity. We sought to interview at least 20 users and five partners, to centre users’ voices but also incorporate partner perspectives. We also aimed to interview at least five users in each of the groups: ages 18–29 and 30–44, never/ever pregnant before, and no experience using *Natural Cycles* in ‘prevent’; and at least ten who *had* used ‘prevent’ to explore switching between modes. We balanced efforts to achieve theoretical saturation (when no new themes emerged in the data) (Charmaz, 2015) against recruitment opportunities and resource availability.

Participants completed an online or PDF consent form in advance. We conducted interviews via videoconferencing software (Skype or Zoom), occasionally without a webcam and/or moving to phone because of connectivity problems. Before beginning, the interviewer confirmed with participants that they were in a private, comfortable space. Interviews with users and partners were undertaken separately, although a few participants invited their partner into the conversation briefly. Interviews lasted on average 48 minutes (25–72) and participants received a £40 voucher in appreciation of their time.

We developed a topic guide to explore: conception‐related timing, planning and events, intentions, expectations and ambiguities; how participants and their partners accessed, experienced, viewed and talked about *Natural Cycles*, other apps and methods (including with healthcare professionals, friends and family); how this shaped their relationships, sexual practices and fertility‐related knowledge; views on the app’s cost, marketing and accessibility; and access to fertility‐related information and care. We used the guide flexibly to follow participants’ narratives while covering key topics of interest, adapting it throughout data collection as additional questions emerged.

Interviews were audio‐recorded with consent and transcribed verbatim. We removed names and potentially identifying biographical details from transcripts. We stored consent forms, anonymised responses to screening/eligibility questions and participants’ contact details securely, separately from each other and from interview data, in accordance with the UK Data Protection Act (2018). After each interview, we debriefed and wrote field notes about the process, dynamics, emerging themes and gaps, reflecting also on how our interests in gender, sexuality, health and social justice shaped the data generated. We analysed data inductively and iteratively, reviewing early transcripts to inform subsequent interviews, sampling and coding. We coded data using an iterative process similar to moving from ‘open’ to ‘axial’ and ‘selective’ coding in grounded theory (Charmaz, 2015), managed in *NVivo 12* qualitative analysis software. Through this analysis, we identified four thematic areas: (1) ways of knowing, at the intersections of ‘nature’, ‘science’, technology and the body; (2) affective intervention of technology into daily lives and relationships; (3) gendered structure, control and responsibility; and (4) silences, support and inclusion/exclusion related to fertility awareness. We use double inverted commas to indicate interview extracts and single ones to denote concepts, categories and other authors’ quotations. We use pseudonyms to maintain participants' anonymity.

The study received ethical approval from the London School of Hygiene and Tropical Medicine Ethics Committee (ref: 16141).

## 3 Findings

We interviewed 24 *Natural Cycles* ‘users’, all cisgender women, aged 24–43 (17 aged 30+, 1 aged 40+); three of whom described their ethnicity as African, Black, Caribbean or Black British; three as Asian or Asian British; two as mixed or multiple ethnic groups; and 18 as white (16 as white British). All women had a current partner; one of these partners was female and the remaining were male. Most (n = 17) were employed full‐time and ‘comfortable’ or ‘really comfortable’ (n = 15) on their current income (a minority were part‐time, self‐employed, studying or retired; nine ‘neither struggling nor comfortable’ or ‘struggling’ on income; none ‘really struggling’). Most participants had used *Natural Cycles* in ‘prevent’ (n = 17) and were currently using ‘plan’ (n = 18). However, this did not always align neatly with pregnancy expectations: some had used ‘prevent’ to build up data for planning rather than as contraception; a few began using ‘plan’ to monitor their cycles before explicitly seeking to conceive. Women had used ‘plan’ for varied durations, from less than a month (n = 4) to almost two years (13 for 1–6 months; seven for over six months). Seven had been pregnant before and six were pregnant when completing eligibility questions, some having switched to ‘follow a pregnancy’ mode or stopped using the app. We interviewed six ‘partners’, all cisgender men, aged 30–39, white British, and most (n = 4) ‘comfortable’ or ‘really comfortable’ on their current income (two ‘neither struggling nor comfortable’). All described their partner as currently using ‘plan’, but at interview some had become pregnant and switched to ‘follow’ or stopped using the app. We refer to women and men to indicate participants interviewed as ‘users’ and ‘partners’, respectively. In practice, ‘user’/‘partner’ categories were not mutually exclusive (see below).

### 3.1 Ways of knowing

Notions of nature and science were central to how participants talked about what *Natural Cycles* meant to them, why and how they used it, and how they came to *know* their bodies. The sense that the technology is both *natural* – compared with the biomedical intervention of hormonal contraception and fertility treatments – and *scientific* – as opposed to traditional methods – made it appealing to prevent and plan pregnancy; a science that could "*feed the natural process along*" (Samira). Women often linked these preferences to their own experiences of hormonal contraception side‐effects (e.g. weight gain, acne, mood changes, depression), and others’ accounts of the physical and emotional toll of *in vitro* fertilisation (IVF) alongside desires to conceive "*naturally*":

I just didn't like the idea of pumping myself full of…chemicals…I wanted to use something as natural as possible…I've never…wanted to use the rhythm method per se because I didn't really trust it, but I thought this seemed a bit more scientific…being able to check your temperatures and all of that stuff. (Samira)

We’re now on a list for IVF…I’m sort of like, ‘Argh, tried to do everything not to get to that point, to just try and do it naturally’. (Joanne)

While some participants recognised that monitoring temperature was not a new method of fertility‐tracking, *Natural Cycles* was widely considered modern and scientific; credible because it was *data‐driven*, "*proven*" (Jessica) and "*endorsed*" (Adina) by well‐known health bodies. Contrary to private pharmaceutical companies "*selling you a drug*" (Samira), *Natural Cycles* was often seen as a small, well‐intentioned business that allowed users to manage their own data about their body and was trusted not to misuse their data. Some participants doubted its accuracy (see below), one after being told by her general practitioner (GP) that BBT was not a reliable indicator of ovulation. However, others used the app to visibly demonstrate to healthcare providers how long they had been trying or the timing of conception, the latter generating surprise at its apparent precision.

Most participants appreciated the app for its straightforward use. The graph was a highly popular means of visualising temperature and ovulation predictions over time. Some liked its reliance on this one measure, as opposed to a wide range of signs that other fertility awareness apps tracked (e.g. mood, cervical secretions, pain). However, others recorded this information within *Natural Cycles* and would have liked it to be incorporated into predictions. The colour gradient in ‘plan’ – more intense red indicating increasing fertility – was a welcome addition to some participants but lacked clarity for others. Some considered the use of green versus red "*counterintuitive*" (Samira), given associations of green with ‘nature’ and ‘fecundity’ and red with ‘danger’, but this was felt to be easy to get used to. Some women appreciated that the technology did not use colours or symbols stereotypically associated with femininity (e.g. flowers).

Women frequently described the app helping them to get to know their bodies and cycles in a way that they had never been taught about, or paid attention to, previously, or that had been obscured by long‐term hormonal contraception:

I was the kind of person that didn’t really pay any attention to when my period would be…it’s like shown me…how long my cycle is, when I’m likely to ovulate…it’s actually made me a lot more in‐tune with myself…now I generally know roughly when I’ve ovulated, whether or not I’ve been using the app consistently. (Sahar)

Indeed, some longer‐term users became so attuned to their cycle that they no longer needed the app. For some, it had also helped their partner become more aware of their cycle and related pain, emotions and mood. Nevertheless, participants often attributed the app’s quality to quantified, disembodied data (temperature readings) and algorithms. Acts of measuring and entering data, and viewing them graphically, allowed users (and sometimes their partners) to approach conception as a project. Visible, disembodied information could offer "*proof*" (Amy) of invisible bodily processes, and explain moods, feelings and energy levels, to women and their partners―amid broader societal dismissal of women’s health concerns, emotions and embodied knowledge:

If he didn’t believe something I was saying…like, ‘my window’s so short’…I showed him the app and he was like, ‘Oh, okay, I get it…if the app’s saying it then it’s got to be correct.’ (Amy)

She does get a little bit moody just beforehand and sometimes it’s quite good to…have a little look…‘Oh it’s just that…nothing I’ve done wrong.’ (Derek)

Using the app also involved generating and interpreting disembodied data through bodily processes and interactions with the technology; *doing* and *seeing* the science. Furthermore, it did not always preclude qualitative, embodied ways of knowing. Some women knew when they ovulated because of cervical secretions, pain and/or feeling warmer ("*I know what it feels like to ovulate"*, Kara). For some, the app prompted them to notice and monitor these sensations; for others, it offered information that complemented or contradicted bodily sensations, leading them to accept or reject the app’s reliance upon temperature, respectively:

Knowing the signs…when you’re ovulating…cervical mucus…things like that…combined with the temperature and seeing it on the graph, it’s like, okay, a bit more of an accurate reading. (Marie)

For me the temperature thing didn’t work, but I still ovulate every month…they should include mucus, that’s the main sign that you need. (Cleo)

Most women also used ovulation strips, generally prompted by the app, often buying cheaper versions online than those available to purchase from *Natural Cycles*. One participant considered these vital to the app’s functionality, but others dismissed them when they contravened temperature‐related predictions, or their own feelings of when they were ovulating.

### 3.2 Affective intervention into daily lives and relationships

Users and partners often framed *Natural Cycles* as a less obtrusive alternative to biomedical intervention. Yet the daily act of entering and viewing data, immediately after waking, rendered this *technological* intervention highly present in many users’, and some partners’, lives. Constant smartphone access could place the app, and fertility tracking, at the centre of daily actions and thoughts ("*I thought about it [my cycle] more continuously…because it was on my phone and I could kind of mess around…see it*", Cleo). Although participants mentioned other influences on fertility – such as (ir)regular cycles, polycystic ovary syndrome (PCOS), endometriosis, age, diet, alcohol and physical activity – they often talked about the app as integral to conception ("*I didn’t expect it…a month [after starting ‘plan’]…the quality of the app I suppose",* Laura; "*It hasn’t got me pregnant yet!"*, Sahar). Indeed, one woman attributed her contrary position to already having a child, compared with those whose desires to conceive were more urgent:

I don't think I’m so wedded to the app…it's there to help me, I'm not angry at it because I'm not pregnant, I'm not relying on it to get pregnant entirely…it's just sort of there…there are probably some people who are much more…obsessed with it because they're obsessed with wanting to get pregnant. (Samira)

Daily app use could offer the reassurance of "*knowing what’s happening…*[and that]…*I’ve done everything possible*" (Victoria), contrary to popular, and often frustrating, advice to "*just relax*" (Amy). It helped keep track of cycles, and for some timing and frequency of sex, amid busy lives, giving users one less thing to worry about. Some women expressed an affinity with an app that was "*getting to know*" (Marie) their bodies, feeling excitement upon opening it, viewing predicted ovulation and ‘peak fertility’ dates. For others, especially those using ‘plan’ for longer, these practices and events were a constant reminder of "*failed*" (Hannah) cycles and unfulfilled expectations. Slight fluctuations in temperature could generate stress and anxiety over not conceiving and, for those who had conceived, possible early miscarriage:

Sometimes I think using the app has made me more stressed about trying to conceive…before my period comes I’m literally thinking, ‘Don’t drop [temperature], like really don’t drop today…’ and then it does and you’re like ‘urgh!’. (Sahar)

When I was pregnant…one of the things that popped up and it scared the living daylights out of me was that, ‘If we see your temperature decreasing that could be the signs of an early miscarriage’…at that point I freaked a little bit…‘Shit, has it decreased by like 0.01 degree?’…I lost it at six weeks. (Emily)

Some participants adapted their app use to manage this, restricting how often they opened it, or taking breaks from measuring their temperature during or between cycles. Samira even attributed her less consistent use to deferring worries about not conceiving: "*I can use the excuse of, ‘Oh well I'm not using it very well’*".

Using the app also had implications for sexual and emotional intimacy, whether or not participants felt it affected when or how often they had sex. For some, it had introduced a shared sense of fun and excitement:

I rolled over and put my temperature in and it popped up with ‘extremely fertile,’ and I straightaway showed that to my partner and within moments we did the deed…That was really fun…to share that experience and the ‘what’s going on with my body?’ (Jessica)

For others, it strained their relationships and routines, and put pressure on them to have sex within a prescribed timeframe – which could change as predictions altered – making it more "*functional*" (Joanne) and less "*romantic*" (Samira):

You wake up in the morning and look at it and go, ‘Okay, brilliant, I’m on my peak fertility…’ So you make that extra effort that night…a few days later I’ll look at it and suddenly that’s my peak day…for whatever reason it [sex] doesn’t happen…my poor husband is like, ‘…I love this, it’s great, but I kind of feel like you’re just using me a couple of days a month and that is it.’ (Emily)

Using *Natural Cycles* could also disrupt spaces of intimacy and sleep, because of the presence, sounds and lights of phones and thermometers in bedrooms. Some women accommodated these pressures and disruptions, for example altering how they talked to their partner ("*It comes up with like ‘peak fertility’…but I wouldn’t exactly use those words…it’s a bit off‐putting, isn’t it?*", Sahar) or moving to another room to take their temperature:

I wake up, I go for my morning pee and I put the thing in my mouth…I do know that you should do it…when you’re still in bed but it’s apparently too loud for my boyfriend…it wakes him up…I find that…a bit annoying because I would probably prefer to do it in bed, also it would probably be better results. (Michelle)

Others described feeling guilty or sorry for their partner for dictating when sex would/not happen, and for asking them to adapt their routines or diets. This can thus be understood as a form of gendered (emotional) labour, as women reworked how they interacted with and talked about the technology to accommodate their partner’s needs and feelings, at a practical and sometimes emotional cost to themselves.

### 3.3 Gendered structure, control and responsibility

Many women described the app – and the knowledge they gained through it – as affording them a new level of control over their bodies and fertility. They contrasted this with medically circumscribed contraceptive choices and socio‐cultural expectations to let chance dictate conception timing. Some participants appreciated that *Natural Cycles* was developed by a woman based on her own experiences, and one even wondered if objections to the app stemmed from concerns that they give "*quite a lot of power over to the female body*" (Laura). Participants often started to use ‘plan’ at the "*right time*" (Nicola) and place in their lives, when they had stable relationships, jobs and living situations; some having used ‘prevent’ to prepare for trying to conceive, and/or at a time when pregnancy "*wouldn’t have been the end of the world*" (Jessica). Whether switching to ‘plan’ was a pivotal moment or a fluid process depended on the relative (un)certainty women and their partner felt about conceiving at that time – contingent upon broader life pressures and aspirations. Some of the women we interviewed were highly conscious of how their age might affect their fertility. This awareness heightened pressure and engagement in practices, including use of technologies, to increase chances of conception: "*you kind of feel like it’s now or never*" (Laura). Age‐related timing was also mentioned by partners, in terms of how they saw themselves as prospective parents: "*I don’t want to be like an old dad, so I think now is the time, like I really want it now*" (Derek). Many participants also talked about becoming increasingly aware of how long it might take to conceive. Amara described how using the app helped to have some control over this anxiety:

I think now that particularly my generation of friends who are going through the process, I’ve got a number that have babies, a number that are trying, you start to realise then how long it can take to conceive and have children and yeah, it is, it is something that does concern me a lot. But I think the app kind of helps as much as it can, like me knowing every day where I’m at in terms of my cycle. I think that has to help the process. (Amara)

Closely connected to the sense of control and autonomy that the app afforded women was that of responsibility: that it is up to the user to engage in the technology "*properly*" (Lisa). Using *Natural Cycles* demanded a "*strict regime*" (Laura) of taking one’s temperature immediately upon waking, at a similar time each day, after at least three hours of rest and no alcohol consumption the previous night, with minimal bodily movements. Monitoring temperature needed to become habit, done consistently, including through reminders (e.g. setting alarms, placing thermometer on mobile phone, as recommended by the company). This was relatively achievable for participants with an established routine, but less so for those with varied work schedules or sleep patterns, for example participants who had children or who travelled frequently. Even slight disruptions could affect precision, which sometimes raised doubts over the technology but also caused participants to question if they were using it ‘right’:

It [temperature] just was completely, like up and down all the time, it seemed to depend where I put it in my mouth, when I woke up…I wake up between half‐six and seven every day, but my partner lives half the week in [a different city]…so that affects…my body temperature…I don’t know what it’s to do with it…maybe I kind of move too much or something…you’re not really supposed to move before you take your temperature. (Cleo)

Certain features of the app itself also directly reinforced user responsibility – for example, rewarding temperature data entry and indeed positive pregnancy tests as ‘achievements’.

Not everyone trusted the app for contraception, particularly those who had not used ‘prevent’, or the few who (knew someone who) had conceived while doing so. However, participants rarely placed sole responsibility on the technology; they also blamed themselves ("*I felt incredibly stupid for having put my faith in an app"*, Christopher) and others for overly depending on it:

Say you’re not in a relationship…you’re being let’s say irresponsible, that’s your own fault…you need to be using additional protection, you can’t rely on an app because your body does change throughout the month. (Emily)

Partners’ involvement varied considerably. Although the decision to begin using the app was sometimes shared, it was typically―albeit not exclusively―women who researched, initiated and engaged in its day‐to‐day use, in addition to wider research online and negotiating discussions with family and friends (see below). Few participants were aware that partners could download the app and share their data if users wished. Thus, this fertility awareness app placed responsibility on women in similar ways to existing methods:

I've often wondered…contraception is still heavily sort of feminised…here was another version of the same thing…about women's responsibility to get pregnant or women's responsibility to not get pregnant. (Samira)

Some women linked their partner’s lack of involvement in using *Natural Cycles* to mistrust, preference for chance and spontaneity, and concerns for her emotional wellbeing, but others to a lack of interest, understanding of "*the personal investment*" (Michelle), or willingness to share responsibilities ("*He possibly wouldn’t be that interested…it’s hard enough to try and get shared shopping"*, Amanda). When partners were involved, this was usually intermittent and often involved women appealing to their interest in technology, data and/or the timing of sex. Some users and partners were happy with their current arrangements ("*He loves to hear if the app’s telling me stuff, he’s so into it",* Samantha) but others voiced a desire for greater balance. For a small minority of women, their partner shared responsibilities for entering temperature data ("*If we forgot to do a reading it was…both of our responsibilities…[I] really appreciated that…so much of the time…partners don’t even ask*", Lisa) and in one case, for David who was the main user, out of an interest in "*stats*" and "*big data*" relative to his partner’s indifference. Derek suggested the app should be more widely marketed to partners, but not everyone desired this level of involvement, as some women felt that sharing the app could afford their partner greater access to their information than they would like.

Interviews also highlighted broader responsibility to research and society. Few participants were worried about data privacy – in part because they trusted *Natural Cycles* to protect their data or felt these metrics gave little information about them, although Cleo described using the app as "*giv[ing] away your power a bit…because that way it was tracking me*". Indeed, some participants considered using the app – and taking part in research – an opportunity to contribute to knowledge generation, support *Natural Cycles* and help others trying to conceive.

### 3.4 Silences, support and inclusion/exclusion

Participants linked difficulties in talking about fertility partly to social pressures to have children; but also to silences and shame around women’s bodies and fertility, reflected in education, religious and popular discourse:

I went to a Catholic school…having come from…the kind of culture where it’s…‘Shh…have your tampons and your pads and go and deal with it quietly.’ (Hannah)

School petrifies you about everything if you don’t want a baby, and then you have to retrain your whole body when you’re thinking, ‘now I want kids’, because no‐one ever teaches you anything about that. (Samantha)

This silence could be particularly isolating for women who were worried about conceiving or had experienced miscarriage. Some participants preferred to talk only to their partner about trying to conceive, but for those who *did* talk to others, this required close friends, family or colleagues who they could talk to without pity, judgement or pressure, facilitated by shared experience. Samantha felt that the "*modern*", technological nature of the app made it easier to initiate conversations about fertility, amid increasingly open discussions about sex and health:

Having an app on your phone, it’s like cool and new and people are more down to talk about stuff like that…generations now talk about contraception, sex, wellbeing, health, much more now than they ever have before…but definitely people that have an app are more open and honest to have a chat about it. (Samantha)

A few women had received helpful fertility‐related advice from GPs, including recommendations to track their cycles (one first having heard about *Natural Cycles* this way). However, most had not talked about trying to conceive with healthcare professionals. Some did not feel the need, but others avoided doing so, after feeling dismissed over concerns about contraceptive side‐effects and fertility, or unjustified in bothering an over‐stretched health service. Apps, and online resources such as NHS websites, were thus important sources of fertility‐related information.

Participants highly valued what they had learned while using *Natural Cycles* and generally considered the messaging appropriate, informative and accessible. However, euphemisms (e.g. "*Get between the sheets!*", Emma; "*preggo"* (Andrew) to mean pregnant), while popular with some, felt vague or insensitive to others. Some participants found online discussion fora, including communities of *Natural Cycles* users, reassuring and informative but others avoided them because they exacerbated their anxieties over conception and pregnancy. While some women felt apps could help them "*deal with your emotions on your own*" (Samantha), others voiced concerns about a lack of meaningful support, particularly for people trying to conceive for an extended period. Indeed, various participants urged *Natural Cycles* to offer more reassurance, information and advice on when and how to seek further care:

In the ‘plan’ mode, I think it might be helpful that there was just a bit more general information about trying to conceive…how long it might take, what’s normal…what the cervical fluid should look like…when you get your period, like it might be nice for the app to say…‘Oh that’s okay…it can take up to like a year’…because you do find yourself getting a little bit…frustrated…it’s nice to be reassured. (Sahar)

Interviews also raised questions over who the technology was intended for. Advertisements appeared to prioritise the ‘prevent’ mode, reflected in language, imagery and actors’ ages. Some participants also noted that women of colour, sexual and gender minorities, and women with disabilities were not well represented, creating an image of typical users as white, heterosexual, non‐disabled, cisgender women:

I haven’t seen women of colour on these ads…there’s no…woman in a wheelchair’ (Amy)

Like anything to do with baby‐making, it’s…very heteronormative… I just find it…quite interesting, that a kind of modern app…it’s made for a woman, some sort of 29‐year‐old in a heterosexual relationship, who’s very steady…in the advertisement…they’re both white…this wholesome kind of Natural Cycles thing, which I guess…goes a bit with…Swedish national image. (Cleo)

Kara’s account – that she could "*relate with it more because there was a black lady in the ad*" – highlights the significance of seeing oneself represented and points to changes to *Natural Cycles* marketing in the UK in recent times. Most participants considered the app reasonably priced, its cost sometimes inspiring trust reflective of credible research and development. Some budgeted to cover the cost, but most noted their privileged financial status and acknowledged that the app would be less affordable to those on low incomes. Some women had switched to ‘follow’ mode while their subscription remained valid, but most felt that it did not offer features beyond other free pregnancy apps.

## 4 Discussion

Participants widely viewed *Natural Cycles* as a scientific yet natural tool to support planning pregnancy – one that could teach them about their bodies and help make sense of, but not necessarily replace, felt, embodied knowledge. It could reassure but also disquiet, particularly for those trying to conceive for longer, while variously introducing excitement, pressure and tension into relationships and spaces of intimacy. Although many women felt the app gave them control over their bodies and fertility, this came with gendered responsibility and labour to engage in the technology with discipline, albeit not overly dependently. A few partners shared in this process, at times out of interest in technology but sometimes also concern for greater equity. Cultural silences around fertility could, in certain circumstances, be disrupted by talking about shared experience and/or technology use. Some participants felt unable to seek fertility advice from NHS health professionals, amid austerity pressures and prior dismissal of their concerns. Apps could partly fill this gap, but there were concerns about such technologies when not connected to human and medical support.

Lupton (2015b) argues that a ‘*valorisation*’ of quantified self‐tracking continues to limit space for women’s embodied experiences. Similarly, Mittelstadt *et al*. (2016) contend that decisions that have in the past been left to humans, such as when to start trying to conceive, are increasingly being delegated to algorithms. With time and continued use, personal analytics transform and quantify the human body (Zampino, 2019). Yet self‐tracking also inevitably involves embodied actions and sensations, as users interact with, relate to, talk about and resist apps and linked objects (Chen, 2017). Many participants voiced trust in visible, disembodied, quantitative metrics to interpret their bodies and ‘*moods’*. However, some emphasised what bodily processes and sensations told them, and believed these over apps (and health professionals) in certain circumstances, thus (re‐)centring embodied and intuitive knowledge (Chen, 2017).

Participants’ accounts also highlighted *affective* dimensions of using fertility awareness apps. Proponents, including many users, praise apps as inobtrusive alternatives to hormonal and barrier contraception (Hodgson, 2018). Yet our findings demonstrate how these technologies intervene, materially, socially and affectively, into the spaces of users’ everyday lives and relationships (Chen, 2017), with positive and detrimental effects. Participants managed negative effects by reworking their use of the technology, and accommodating their partners’ needs and views, but this typically required their (emotional) labour. Furthermore, while pleasure can be gained from self‐monitoring via apps (Chen, 2017), users often adapt and are selective over what is recorded (Gorm and Shklovski, 2019, Weiner *et al*., 2020), this ‘*curation’* affording what Weiner et al., (2020) argue constitutes a way to *‘support their motivation and protect themselves from disappointing outcomes’*. Ruckenstein and Dow Schüll, (2017) hold that this volatile relationship can shift users’ emotional engagement with such apps from *‘hope to disappointment, pleasure to frustration, control to obsessiveness’*. Women have reported frustration, disappointment, disillusionment, and undermined agency when apps do not function ‘*as intended*’ (Lupton and Maslen, 2019), and becoming ‘*consumed*’ with tracking fertility (Gambier‐Ross *et al*., 2018). Despite growing concerns around links between social media use and mental health (Igor, 2014), public health research has focused more on self‐tracking apps designed to manage mental health (Donker *et al*., 2013) than the emotional *processes* and *effects* of using them – a gap that we believe warrants further attention. The pressures and anxieties that users may experience while trying to conceive and using apps require acknowledgment. Most young people report intentions and expectations of having children in the future but tend to over‐estimate chances of conception (Prior *et al*., 2019). For many, the ability to have children affects how they feel ‘valued or devalued as members of society’ (Faircloth and Gurtin, 2018). As trends towards later motherhood and age‐related fertility declines gain greater attention, reproductive technologies to aid conception are becoming more widely adopted. However, with increased knowledge gained through technology and monitoring comes a greater, usually highly gendered, burden (Chen, 2017), as women often bear primary responsibility for ‘fertility management’, including the timing of sex and other pre‐conception practices, investigations and treatments, and ultimately parenthood (Baldwin, 2019).

In our study, self‐tracking allowed, and required, participants to be responsible ‘*digitised, reproductive citizens*’ (Lupton, 2015a) who use technology ‘*properly*’, organise their lives accordingly and improve self‐ and collective knowledge, amid commercial technological innovation. Some explicitly voiced their support of *Natural Cycles* as a growing, female‐owned business. However, any discussions over how such technologies can ‘*revolutionise*’ women’s reproductive health must consider the continued responsibility they place on women using them, how accessible they are, and their potential co‐optation to restrict birth control options (Bryant, 2019). Our findings also demonstrate how marketing, functionality and cost shape notions of who such technologies are ‘for’. Amid financial pressures on health systems, interest in incorporating online and mobile health interventions into service delivery is growing (Free *et al*., 2013, Labrique *et al*., 2013). *Natural Cycles* and other fertility awareness apps suit some people whose lives and incomes accommodate their use, and their incorporation into health service delivery may remove costs to end‐users. However, critical questions remain as to how such technologies may exacerbate broader health and social inequalities (Lupton, 2016), including in relation to class, race, disability, gender and sexuality.

Some women felt that silences around fertility were slowly being disrupted by generational shifts and technological advances. Other researchers link willingness to share fertility tracking data to the diminishing ‘taboo’ of talking about menstruation, although doing so with health professionals functioned to be ‘*taken seriously*’ rather than facilitating talk on women’s own terms (Gambier‐Ross *et al*., 2018). Participants did not universally wish to talk about their app use with others, but digital technologies did provide them with knowledge they had not acquired through education and healthcare interactions, and sometimes stimulated broader discussions with friends and family. In the UK, where school curricula and health promotion focus heavily on avoiding sexually transmitted infections and pregnancy, fertility awareness remains low (Harper *et al*., 2017). GPs and practice nurses are restricted in their capacity to provide related counselling by short consultations and inadequate training (Hampton *et al*., 2016). Meanwhile interventions typically target couples once they are having difficulties conceiving rather than when they begin trying (Hvidman *et al*., 2015). There is a clear need for schools, healthcare spaces and media to facilitate more open discussions around fertility (Harper *et al*., 2017).

This is the first qualitative study to explore how cisgender women and partners use apps while trying to conceive. We offer a critical account of how technologies hailed as novel, liberating and non‐invasive can nevertheless reproduce entrenched gendered responsibilities for conception and emotional labour, as well as broader digital health inequalities, building on existing literature surrounding reproductive citizenship. Our findings expand knowledge of how fertility awareness apps, despite being understood as ‘natural’ alternatives to biomedical fertility treatments, intervene in intimate, affective, bodily and other spaces, and personal relationships, on a daily basis, through assemblages of related technologies and objects (e.g. mobile phones, thermometer, algorithms and graphs). While we observed the privileging of disembodied, quantifiable data that Lupton (2015) and others have described, we also found that women’s bodily and affective engagements with the technology and, at times, continued reliance on felt, bodily understanding allowed for a certain (re‐)centring of embodied knowledge (Chen, 2017).

Our focus on a pay‐app, medically accredited as a contraceptive, limits generalisability to other fertility awareness apps. However, using a critical digital health studies lens provided valuable insights into participants’ technology‐related practices relative to their everyday lives, relationships, bodily and social processes, norms and power relations. We may not have reached users less engaged with the app or those with less positive experiences (participants were self‐selecting and most learned of the study through *Natural Cycles*) but participants voiced varied perspectives and some had stopped using it. This is the first qualitative study to include partners, but all six were white, cis‐male, in their 30s, and most were financially comfortable and did not have children. Although we interviewed a more racially‐, economically‐ and age‐diverse range of users, just one participant had a female partner and no participants identified as transgender or non‐binary; although our language was implicitly inclusive, we did not ask users about their own gender identity in sampling questions, meaning that we could not purposefully select participants on this basis. One participant told us she had disabilities, but it is by chance that she disclosed this as we did not ask explicitly about this. In future studies, we would urge researchers to purposefully include women with female partners, transgender and gender non‐conforming people, individuals with disabilities and those with less financial means, as well as participants of varied ethnicities and ages, to better understand how such technologies cater to their needs, bodies, identities and lives.

## 5 Conclusion

Technology has the potential to afford women a sense of control and knowledge over their fertility, amid a lack of related education and financial pressures on health services. *Natural Cycles* users generally appreciated the technology as a ‘scientific’ means of supporting and learning about conception in a way that still felt ‘natural’. However, daily monitoring can also exacerbate anxiety, and pressure on sex, relationships and communication between partners, while reinforcing gendered labour and responsibility for fertility. Any efforts to roll out fertility awareness apps must attend to what such technologies can and cannot achieve, including how they may reinforce existing gendered, racial, economic and other power imbalances. To avoid worsening existing health inequalities, it is crucial that app design, health promotion materials and future research value potential users’ embodied knowledge and are actively inclusive and accomodate their diverse lived experiences, identities and needs.

## Author contribution

**Pippa Grenfell:** Conceptualization (supporting); Data curation (lead); Formal analysis (lead); Funding acquisition (supporting); Investigation (lead); Methodology (equal); Project administration (supporting); Resources (supporting); Software (lead); Supervision (supporting); Validation (equal); Visualization (equal); Writing‐original draft (lead); Writing‐review & editing (equal). **Nerissa Tilouche:** Data curation (supporting); Formal analysis (supporting); Investigation (supporting); Methodology (supporting); Project administration (equal); Resources (equal); Software (supporting); Writing‐original draft (supporting). **Jill Shawe:** Conceptualization (supporting); Data curation (supporting); Formal analysis (supporting); Funding acquisition (supporting); Investigation (supporting); Methodology (supporting); Supervision (supporting); Validation (supporting); Writing‐original draft (supporting); Writing‐review & editing (supporting). **Rebecca French:** Conceptualization (lead); Data curation (supporting); Formal analysis (supporting); Funding acquisition (lead); Investigation (supporting); Methodology (equal); Project administration (supporting); Resources (equal); Supervision (lead); Validation (supporting); Visualization (equal); Writing‐original draft (supporting); Writing‐review & editing (equal).

## Funding

This study was undertaken with funding from *Natural Cycles*. The funder had no involvement in study design, data collection, analysis or interpretation, including the decision to publish. The views expressed in this publication are those of the authors and not necessarily those of *Natural Cycles*.

## 6 Competing interests

None of the authors have any involvement in *Natural Cycles* and they did not receive any financial or other incentive to conduct this research other than their usual university salaries. Before developing the Freyja Study proposal, Dr Rebecca French and Prof Jill Shawe each received an honorarium from *Natural Cycles* to participate in an expert workshop aimed at identifying research gaps in relation to fertility awareness methods.

## Data Availability

Research data are not shared.
